# Association between the Melatonin Receptor 1B Gene Polymorphism on the Risk of Type 2 Diabetes, Impaired Glucose Regulation: A Meta-Analysis

**DOI:** 10.1371/journal.pone.0050107

**Published:** 2012-11-30

**Authors:** Qing Xia, Zi-Xian Chen, Yi-Chao Wang, Yu-Shui Ma, Feng Zhang, Wu Che, Da Fu, Xiao-Feng Wang

**Affiliations:** Department of Orthopedic Surgery, Zhongshan Hospital, Fudan University, Shanghai, P.R. China; Innsbruck Medical University, Austria

## Abstract

**Background:**

Melatonin receptor 1B (MTNR1B) belongs to the seven-transmembrane G protein-coupled receptor superfamily involved in insulin secretion, which has attracted considerable attention as a candidate gene for type 2 diabetes (T2D) since it was first identified as a loci associated with fasting plasma glucose level through genome wide association approach. The relationship between *MTNR1B* and T2D has been reported in various ethnic groups. The aim of this study was to consolidate and summarize published data on the potential of MTNR1B polymorphisms in T2D risk prediction.

**Methods:**

PubMed, EMBASE, ISI web of science and the CNKI databases were systematically searched to identify relevant studies. Odds ratios (ORs) and 95% confidence intervals (95% CIs) were calculated. Heterogeneity and publication bias were also tested.

**Results:**

A total of 23 studies involving 172,963 subjects for two common polymorphisms (rs10830963, rs1387153) on *MTNR1B* were included. An overall random effects per-allele OR of 1.05 (95% CI: 1.02–1.08; *P*<10^−4^) and 1.04 (95% CI: 0.98–1.10; *P* = 0.20) were found for the two variants respectively. Similar results were also observed using dominant or recessive genetic model. There was strong evidence of heterogeneity, which largely disappeared after stratification by ethnicity. Significant results were found in Caucasians when stratified by ethnicity; while no significant associations were observed in East Asians and South Asians. Besides, we found that the rs10830963 polymorphism is a risk factor associated with increased impaired glucose regulation susceptibility.

**Conclusions:**

This meta-analysis demonstrated that the rs10830963 polymorphism is a risk factor for developing impaired glucose regulation and T2D.

## Introduction

Glucose homeostasis in healthy individuals is tightly controlled through a complex pathway of regulatory mechanisms involving multiple organs and tissues. Disruption of normal glucose homeostasis and substantial elevations of fasting glucose are hallmarks of type 2 diabetes (T2D) and typically result from sustained reduction in pancreatic beta-cell function and insulin secretion. However, there is substantial variation in fasting glucose levels even within healthy, non-diabetic populations. Approximately one-third of this variation is genetic [1], but little of this heritability has been explained.

Recent genome-wide association studies (GWAS) and meta-analysis have identified genes contributing to the variation of fasting plasma glucose (FPG) levels in populations of European origin [2]–[6]. Two common variants (rs10830963, rs1387153) in the melatonin receptor 1B (MTNR1B) were shown to have moderate effects on FPG levels in nondiabetic individuals with an increased risk for T2D [2], [4]. MTNR1B is the receptor of melatonin which inhibits insulin secretion through its effect on the formation of cGMP [7], [8]. Knock-out mice of these genes demonstrated significantly lower fasting glucose levels [9], [10]. Over the past few years, considerable efforts have been devoted to exploring the relationships between the MTNR1B polymorphisms and T2D. Genetic association studies can be problematic to reproduce due to multiple hypothesis testing, population stratification, source of controls, publication bias, and phenotypic heterogeneity. In addition, with the increased studies in recent years among Asian, and other populations, there is a need to reconcile these data. Therefore, we performed a meta-analysis of the published to establish a comprehensive picture of the relationship between *MTNR1B* and risk of T2D as well as to quantify the between-study heterogeneity and potential bias.

## Materials and Methods

### Literature search strategy

Genetic association studies published before the end of May 2012 on T2D and polymorphisms in the *MTNR1B* gene were identified through a search of PubMed, Web of Science, EMBASE and CNKI (Chinese National Knowledge Infrastructure). Search term were keywords relating to the relevant gene (e.g. ‘melatonin receptor 1B’ or ‘*MTNR1B*’) in combination with words related to T2D (e.g. ‘Type 2 diabetes’ or ‘Type 2 diabetes mellitus’ or ‘non-insulin dependent diabetes mellitus’). Furthermore, reference lists of main reports and review articles were also reviewed by a manual search to identify additional relevant publications.

### Eligible studies and data extraction

The included studies have to meet the following criteria: (1) original papers containing independent data which have been published in peer-reviewed journal, (2) identification of T2D patients according to the World Health Organization criteria, American Diabetes Association criteria, or other standard criteria, (3) genotype distribution information or odds ratio (OR) with its 95% confidence interval (CI) and *P*-value;, (4) case–control or cohort studies. The major reasons for exclusion of studies were (1) overlapping data and (2) case-only studies, family based studies, and review articles.

Data extraction was performed independently by two reviewers and differences were resolved by further discussion among all authors. For each included study, the following information was extracted from each report according to a fixed protocol: first author, publication year, definition and numbers of cases and controls, diagnostic criterion, impaired glucose regulation (IGR) status (impaired fasting glucose and/or impaired glucose tolerance), frequency of genotypes, age, sex, body mass index (BMI), Hardy–Weinberg equilibrium status, ethnicity and genotyping method.

### Statistical methods

Deviation from Hardy–Weinberg equilibrium for controls was examined by χ^2^ tests. Odds ratio (OR) with 95% confidence intervals (CIs) was used to assess the strength of association between the *MTNR1B* gene polymorphism and T2D risk. The per-allele OR of the risk allele was compared between cases and controls. Then, we examined the association between risk genotype of these polymorphisms and T2D susceptibility using dominant and recessive genetic models. Heterogeneity across individual studies was calculated using the Cochran chi-square Q test followed by subsidiary analysis or by random-effects regression models with restricted maximum likelihood estimation [11]–[13]. Random-effects and fixed-effect summary measures were calculated as inverse variance-weighted average of the log OR. The results of random-effects summary were reported in the text because it takes into account the variation between studies. The 95% CIs were constructed using Woolf's method [14]. The significance of the overall OR was determined by the Z-test. Sample size (No. cases ≥1000 or <1000) and ethnicity were prespecified as characteristics for assessment of heterogeneity. Ethnic group was defined as Caucasian (i.e., people of European origin), East Asian (e.g., Chinese, Japanese, and Korean), and South Asian (e.g., Indian, and Pakistani). In addition, sample size, ethnicity, gender distribution in cases and controls, genotyping method, mean age and BMI of cases and controls were analyzed as covariates in meta-regression.

Sensitivity analyses were performed to assess the stability of the results, namely, a single study in the meta-analysis was deleted each time to reflect the influence of the individual data set to the overall OR. Publication bias was assessed using Egger's test [15] and Begg's funnel plots [16]. All P values are two-sided at the P = 0.05 level. All analyses were conducted using the STATA 10.0 (STATA Corporation, College Station, TX).

## Results

### Characteristics of studies

The combined search yielded 72 references. Forty-nine articles were excluded because they clearly did not meet the criteria or overlapping references. Finally, a total of 23 studies were retrieved based on the search criteria for T2D susceptibility related to the *MTNR1B* polymorphisms [2], [4], [17]–[37] (Figure S1). The main study characteristics were summarized in Table 1. There are 16 studies with 51, 552 T2D cases and 92, 618 controls concerning rs10830963 and 7 studies with 14, 874 T2D cases and 17, 703 controls concerning rs1387153. These two polymorphisms were found to occur in frequencies consistent with Hardy-Weinberg equilibrium in the control populations of the vast majority of the published studies. Of the cases, 63% were Caucasians, 31% were East Asians, and 6% were South Asians.

**Table 1:** Characteristics of the studies included in the meta-analysis.

Study	Year	Ethnicity	Cases	Controls	Polymorphism	No. of case	No. of control	Genotyping method
Staiger [17]	2008	German	IFG, IGT confirmed by OGTT	Normal glucose tolerant	rs10830963	287 IFG, 275 IGT	1139	TaqMan
Sparsø [18]	2009	European	T2D, IFG, IGT per WHO criteria	Normal fasting glycaemia; Normal glucose tolerant	rs10830963, rs1387153	1948 T2D, 686 IFG, 665 IGT	4905	Taqman
Rönn [19]	2009	Chinese	T2D per WHO criteria	Normoglycaemic	rs10830963	1165 T2D	1105	iPLEX
Prokopenko [2]	2009	European	T2D patients	Non-diabetic	rs10830963	18236 T2D	64353	Taqman, iPLEX, Illumina chip, Affymetrix chip
Bouatia-Naji [4]	2009	French, Danish	T2D per WHO criteria	Normal fasting glycaemia; Normal glucose tolerant	rs1387153	6332 T2D	9132	Illumina chip
Reiling [20]	2009	Dutch	T2D per WHO criteria	Normal glucose tolerant	rs10830963	2537 T2D	1990	Taqman
Tam [21]	2010	Chinese	T2D per WHO criteria	Normal fasting glucose	rs10830963	1342 T2D	1644	MassARRAY
Liu [22]	2010	Chinese	T2D per WHO criteria	Normal fasting glucose	rs10830963	424 T2D	1908	SNPstream
Simonis-Bik [23]	2010	European	IGT per WHO criteria	Normal glucose tolerant	rs10830963	158 IGT	177	Taqman
Xu [24]	2010	Chinese	T2D, IGR per WHO criteria	Normal glucose regulation	rs10830963, rs1387153	1825 T2D, 1487 IGR	2200	SNaPshot
Hu [25]	2010	Chinese	T2D per WHO criteria	Normal glucose tolerant	rs10830963	3410 T2D	3412	MassARRAY
Kan [26]	2010	Chinese	T2D per WHO criteria	Normal fasting glucose	rs10830963, rs1387153	1912 T2D	2041	Taqman
Rees [27]	2011	Pakistani	T2D per WHO criteria	Normoglycaemic	rs10830963	1678 T2D	1584	KASPar
Dietrich [28]	2011	German	IGT, T2D per WHO criteria	Normal glucose tolerant	rs10830963	103 T2D, 73 IGT	748	TaqMan
Been [29]	2011	Indian	T2D per ADA criteria	Normoglycaemic	rs10830963, rs1387153	1169 T2D	1001	TaqMan
Ling [30]	2011	Chinese	T2D per WHO criteria	Non-diabetic	rs10830963	1118 T2D	1161	MassARRAY
Olsson [31]	2011	Norwegian	T2D patients	Non-diabetic	rs10830963	1322 T2D	1447	TaqMan
Ohshige [32]	2011	Japanese	T2D per WHO criteria	Non-diabetic	rs10830963, rs1387153	2809 T2D	2066	PCR-invader assay
Renstrom [33]	2011	Swedish	IFG per WHO criteria	Normal glucose regulation	rs10830963	964 IFG	3087	OpenArray
Song [34]	2011	Chinese	IFG per WHO criteria	Normal fasting glucose	rs10830963	288 IFG	1742	ARMS-PCR
Tabara [35]	2011	Japanese	T2D per ADA criteria	Non-diabetic	rs10830963, rs1387153	495 T2D	399	TaqMan
Liu [36]	2012	Chinese	T2D per ADA criteria	Normal glucose tolerant	rs1387153	295 T2D	239	RFLP
Walford [37]	2012	American	IFG per ADA criteria	Normal glucose tolerant	rs10830963	6251 IFG	12480	IBC Chip

WHO: World Health Organization, ADA: American Diabetes Association, IFG: Impaired Fasting Glycemia, IGT: Impaired Glucose Tolerance, IGR: Impaired Glucose Regulation, OGTT: Oral Glucose Tolerance Test.

### Association of MTNR1B rs10830963 polymorphism with T2D

**Figure 1:**
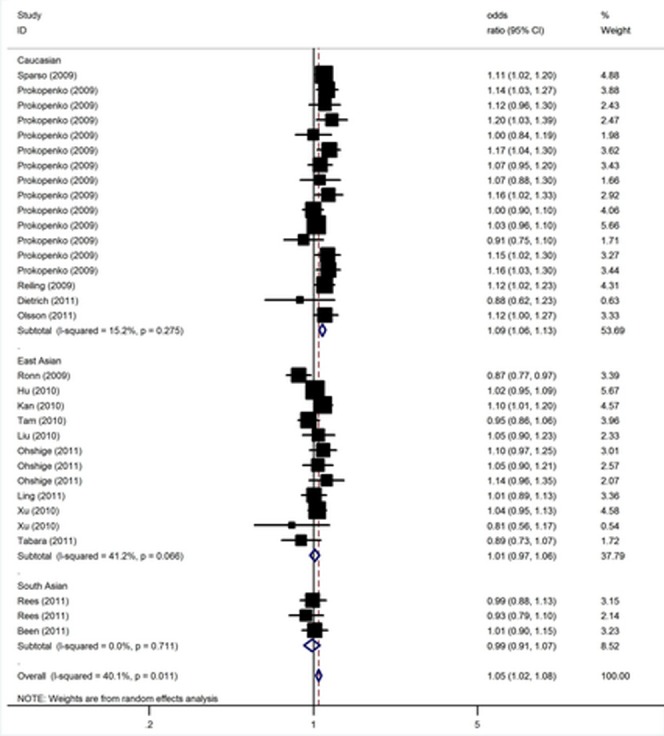
Meta-analysis of studies of the rs10830963 polymorphism of *MTNR1B* and T2D.

Overall, there was evidence of an association between the increased risk of T2D and the variant in different genetic models when all the eligible studies were pooled into the meta-analysis. Using random effect model, the summary per-allele OR of the G variant for T2D was 1.05 [95% CI: 1.02–1.08, *P*(Z)<10^−4^, *P*(Q) = 0.01; Figure 1], with corresponding results under dominant and recessive genetic models of 1.10 [95% CI: 1.06–1.13, *P*(Z)<10^−4^, *P*(Q) = 0.17 ] and 1.12 [95% CI: 1.08–1.16, *P*(Z)<10^−5^, *P*(Q) = 0.007], respectively. In the stratified analysis by ethnicity, significantly increased risks were found among Caucasian populations [G allele: OR = 1.09, 95% CI: 1.06–1.13, *P*(Z)<10^−5^; dominant model: OR = 1.16, 95% CI: 1.07–1.25, *P*(Z)<10^−5^; recessive model: OR = 1.19, 95% CI: 1.10–1.28, *P*(Z)<10^−5^]. However, no significant association was found for East Asian and South Asian populations in all genetic models (Table 2). By considering sample size subgroups, the OR was 1.03 [95% CI: 0.97–1.09, *P*(Z) = 0.32, *P*(Q) = 0.15] in small studies compared to 1.06 [95% CI: 1.03–1.10, *P*(Z)<10^−4^, *P*(Q) = 0.01] in larger studies.

**Table 2:** Results of meta-analysis for *MTNR1B* rs10830963 polymorphism and T2D risk.

Sub-group analysis	No. of data sets	No. of cases/controls	G Allele			Dominant Model			Recessive Model		
			OR (95%CI)	*P(Z)*	*P(Q)*	OR (95%CI)	*P(Z)*	*P(Q)*	OR (95%CI)	*P(Z)*	*P(Q)*
Overall	32	41552/92618	1.05 (1.02–1.08)	<10^−4^	0.01	1.10 (1.06–1.13)	<10^−4^	0.17	1.12 (1.08–1.16)	<10^−5^	0.007
Ethnicity											
Caucasian	17	24146/73443	1.09 (1.06–1.13)	<10^−5^	0.27	1.16 (1.07–1.25)	<10^−5^	0.07	1.19 (1.10–1.28)	<10^−5^	0.04
East Asian	12	14559/16590	1.01 (0.97–1.06)	0.57	0.07	1.06 (0.98–1.13)	0.11	0.09	1.08 (0.99–1.17)	0.07	0.01
South Asian	3	2847/2585	0.99 (0.91–1.07)	0.72	0.71	0.99 (0.88–1.11)	0.81	0.53	1.07 (0.99–1.16)	0.07	0.03
Sample size											
<1000	13	6881/18503	1.03 (0.97–1.09)	0.32	0.15	1.05 (0.99–1.12)	0.11	0.38	1.08 (0.99–1.19)	0.09	0.12
≥1000	19	34671/74115	1.06 (1.03–1.10)	<10^−4^	0.01	1.11 (1.07–1.15)	<10^−5^	0.22	1.14 (1.09–1.19)	<10^−5^	<10^−4^

In meta-regression analysis, sample size (*P* = 0.15), mean age of cases (*P* = 0.28) and controls (*P* = 0.16), mean BMI cases (*P* = 0.06) and controls (*P* = 0.17), sex distribution in cases (*P* = 0.32) and controls (P = 0.21) did not significantly explained such heterogeneity. By contrast, ethnicity (*P* = 0.001) was significantly correlated with the magnitude of the genetic effect, explaining 67% of the heterogeneity.

### Association of MTNR1B rs10830963 polymorphism with impaired glucose regulation

**Figure 2:**
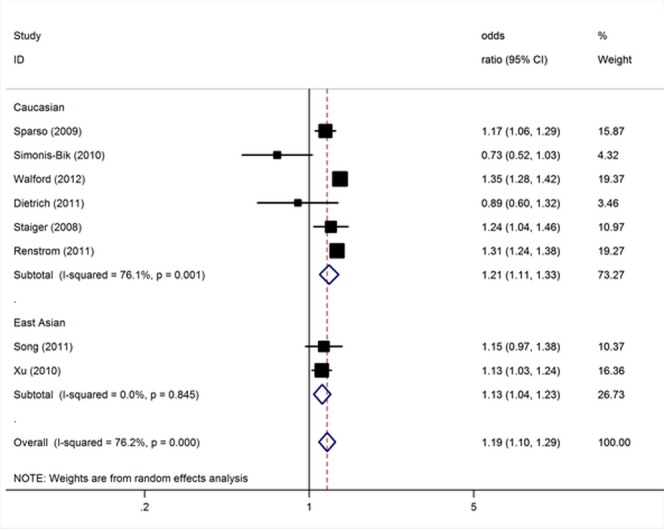
Meta-analysis of studies of the rs10830963 polymorphism of *MTNR1B* and IGR stratified by ethnicity.

To investigate how glucose metabolism was related to *MTNR1B*, we analyzed individuals with impaired glucose regulation (impaired glucose tolerance and/or impaired fasting glucose). The data on genotypes of the rs10830963 polymorphism among impaired glucose regulation cases and controls were available in 8 (including 10, 810 cases and 26, 478 controls) studies [17], [18], [23], [24], [28], [33], [34], [37]. For IGR risk and the rs10830963 polymorphism of *MTNR1B*, our meta-analysis gave an overall per-allele OR of 1.19 (95% CI: 1.10–1.29; *P*<10^−4^) with statistically significant between-study heterogeneity (*P*<10^−4^). Significant associations were also found under dominant [OR = 1.23; 95% CI: 1.15–1.31; *P*(Z)<10^−5^; *P*(Q) = 0.004] and recessive [OR = 1.27; 95% CI: 1.18–1.35; *P*(Z)<10^−5^; *P*(Q) = 0.006] genetic model. This analysis is based on pooling of data from a number of different ethnic populations. When stratifying for ethnicity, an OR of 1.21 [95% CI: 1.11–1.33; *P*(Z)<10^−4^; *P*(Q)<0.001] and 1.13 [95% CI: 1.04–1.23; *P*(Z) = 0.003; *P*(Q) = 0.85] resulted for risk allele, among Caucasians and East Asians, respectively (Figure 2). By considering IGR outcome subgroups, the OR was 1.04 [95% CI: 0.87–1.25; *P*(Z) = 0.66; *P*(Q)<10^−4^] for IGT compared to 1.33 [95% CI: 1.17–1.52; *P*(Z)<10^−4^; *P*(Q) = 0.03] for IGF.

### Association of MTNR1B rs1387153 polymorphism with T2D

**Figure 3:**
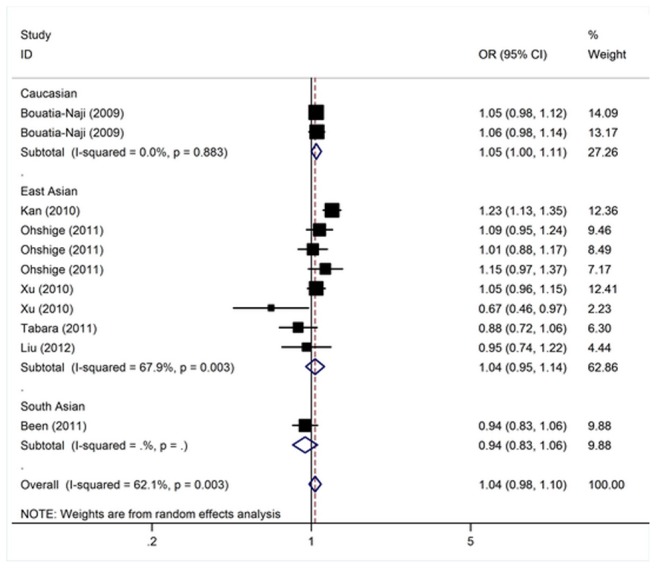
Meta-analysis of studies of the rs1387153 polymorphism of *MTNR1B* and T2D.

The meta-analysis resulted in a statistically non-significant association between *MTNR1B* rs1387153 and T2D. The overall OR for risk T allele was 1.04 [95% CI: 0.98–1.10; *P*(Z) = 0.20; *P*(Q) = 0.003; Figure 3]. Similar results were also found under dominant and recessive genetic models (Table 3). When studies were stratified for ethnicity, marginal significant associations were found among Caucasians with per-allele OR of 1.05 [95% CI: 1.00–1.11, *P*(Z) = 0.048; *P*(Q) = 0. 88]. However, no significant association was found for East Asian and South Asian populations in almost all genetic models (Table 3). Subsidiary analyses of sample size yielded a per-allele OR of 0.96 [95% CI: 0.84–1.10, *P*(Z) = 0.57] for small studies and for larger studies of 1.07 [95% CI: 1.00–1.14, *P*(Z) = 0.04].

**Table 3:** Results of meta-analysis for *MTNR1B* rs1387153 polymorphism and T2D risk.

Sub-group analysis	No. of data sets	No. of cases/controls	T Allele			Dominant Model			Recessive Model		
			OR (95%CI)	*P(Z)*	*P(Q)*	OR (95%CI)	*P(Z)*	*P(Q)*	OR (95%CI)	*P(Z)*	*P(Q)*
Overall	11	14874/17703	1.04 (0.98–1.10)	0.20	0.003	1.03 (0.94–1.13)	0.55	0.005	1.11 (1.00–1.23)	0.05	0.12
Ethnicity											
Caucasian	2	6332/9132	1.05 (1.00–1.11)	0.048	0.88	1.05 (0.98–1.12)	0.14	0.77	1.12 (0.99–1.25)	0.06	0.37
East Asian	8	7378/7598	1.04 (0.95–1.14)	0.42	0.003	1.10 (1.00–1.20)	0.05	0.001	1.18 (1.06–1.31)	0.004	0.11
South Asian	1	1164/973	0.94 (0.83–1.06)	0.32	NA	0.91 (0.77–1.09)	0.31	NA	0.93 (0.72–1.20)	0.57	NA
Sample size											
<1000	5	2045/2690	0.96 (0.84–1.10)	0.57	0.06	0.93 (0.82–1.06)	0.28	0.16	1.07 (0.91–1.26)	0.44	0.19
≥1000	6	12829/15013	1.07 (1.00–1.14)	0.04	0.01	1.08 [1.03, 1.14]	0.004	0.01	1.14 (1.05–1.24)	0.002	0.12

NA: Not Available.

In meta-regression analysis, neither sample size (*P* = 0.37), ethnicity (*P* = 0.46), mean BMI of cases (*P* = 0.17) and controls (*P* = 0.29), mean age of cases (*P* = 0.09) and controls (*P* = 0.67), nor sex distribution in cases (*P* = 0.39) and controls (*P* = 0.11) were significantly correlated with the magnitude of the genetic effect.

### Sensitivity analyses and publication bias

A single study involved in the meta-analysis was deleted each time to reflect the influence of the individual data-set to the pooled ORs, and the corresponding pooled ORs were not qualitatively altered (Figure S2 and S3). Begg's funnel plot and Egger's test were performed to access the publication bias of the literatures. The shape of the funnel plots was symmetrical for these polymorphisms (Figure S4 and S5). The statistical results still did not show publication bias in these studies for rs10830963 (Egger test, *P* = 0.38; Figure S6) and rs1387153 (Egger test, *P* = 0.12; Figure S7).

## Discussion

Large sample and unbiased epidemiological studies of predisposition genes polymorphisms could provide insight into the in vivo relationship between candidate genes and diseases. GWAS and meta-analysis have shown that common variation in the *MTNR1B* (rs10830963, rs1387153) locus increases the level of FPG. However, the relationship between these common variations and T2D susceptibility has not been built up yet. This is the first comprehensive meta-analysis examined the *MTNR1B* polymorphisms (rs10830963, rs1387153) and the relationship to T2D risk. Its strength was based on the accumulation of published data giving greater information to detect significant differences. In total, the present meta-analysis combined 23 studies including 48, 278 T2D cases, 10,810 IGR cases, and 119,960 controls.

Our results demonstrated that the rs10830963 polymorphism of *MTNR1B* is a risk factor for developing type 2 diabetes. In the stratified analysis by ethnicity, significant associations were found in Caucasians for the polymorphism in all genetic models. However, no significant associations were detected among East Asian and South Asian populations for rs10830963 and rs1387153 polymorphisms. There are several possible reasons for such differences. Firstly, the frequencies of the risk-association alleles in *MTNR1B* vary between different races. For example, the G allele distributions of the rs10830963 polymorphism varies between East Asian, South Asian and Caucasian populations, with a prevalence of ∼42%, ∼39%, and ∼25%, respectively. Therefore, additional studies are warranted to further validate ethnic difference in the effect of these polymorphisms on TD risk. Secondly, such different results could also be explained by study design or sample size. Besides, other confounding factors, such as age, sex, life style should also be considered. In the stratified analysis according to sample size, significantly associations were found only found for larger studies. Thus, absence of association with type 2 diabetes in small study could be due to insufficient power. Thus, for future association studies much larger sample size will be required.

Melatonin is mainly produced by the pineal gland but is also released from the gastrointestinal tract [38]. As a highly lipophilic circulating hormone, melatonin easily reaches and penetrates all cells and, in addition to controlling circadian rhythm, it has the ability to neutralise reactive oxygen and nitrogen species and activate the immune system [39]. Several studies have shown a link between disturbances of circadian rhythm and metabolic diseases, including diabetes [40], [41], as well as a clear relationship between insulin and melatonin [42]. Two distinct G protein-coupled receptors, MTNR1A and MTNR1B, mediate the effects of melatonin. These two receptors have been found to be produced in human pancreatic islets [42], [43], and the levels of both are upregulated in type 2 diabetic patients [44]. Furthermore, Lyssenko et al. [3] confirmed the presence of MTNR1B in human pancreatic islets and showed increased MTNR1B mRNA expression in carriers of the rs10830963 risk genotype, reporting a negative correlation between MTNR1B mRNA levels and insulin secretion. The recent finding that MTNR1B is expressed in the β-cells implies that the gene variant might affect pancreatic glucose sensing and/or insulin release and thereby glucose tolerance [4]. However, rs10830963 is located in the intronic region of MTNR1B, while rs1387153 is located in the 5′ region. These variants might be involved in regulation of promoter activity, but additional analyses of these SNPs are required to provide clear evidence on their functional consequences. Candidate gene based studies showed that focusing on functionally significant alleles can increase statistical signal and, hence, the power to detect association between presence of rare variants and complex traits. Recently, Bonnefond et al. reported that rare MTNR1B variants impairing melatonin receptor 1B function contribute to T2D [45], implying that the previously observed increased MTNR1B expression may not be causal.

Impaired glucose regulation (IGR) includes impaired fasting glucose (IFG) and/or impaired glucose tolerance (IGT). IGR is also known as intermediate hyperglycemia or pre-diabetes and characterized by high blood glucose concentrations, insulin resistance and impaired insulin secretion. Previous studies have shown that 5∼10% IGT subjects developed diabetes each year, although, some of them could revert spontaneously to normal glucose tolerance [46], [47]. However, few studies were concerned about the association of those GWAS variations with IGR [48]. IFG and/or IGT were predisposed to diabetes; however, whether the IGR and T2DM shared the same spectrum of genetic variations is not well characterized. Here in our study, we found that rs10830963 of *MTNR1B* that are associated with T2D was also conferred the risk of IGR. Our study suggested that IGR might have similar background of susceptible genetic variations. In addition, our results indicated that significantly increased risk of *MTNR1B* rs10830963 polymorphism was found for IGF but not for IGT when stratified by IGR outcome. However, because the IGR included IFG and IGT which may have different genetic etiology [5], [49], more prospectively-designed association studies with large sample size and homogeneous patients are needed in the near future.

In interpreting the results, some limitations of this meta-analysis should be addressed. Firstly, the subgroup meta-analyses on Asian populations are based on a small number of studies with such information available. Nevertheless, the total number of subjects included in this part of the analysis comprises the largest sample size so far. As studies among the Non-Caucasians are currently limited, further studies including a wider spectrum of subjects should be carried to investigate the role of these variants in different populations. Secondly, our results were based on unadjusted estimates, while a more precise analysis should be conducted if all individual raw data were available, which would allow for the adjustment by other co-variants including age, drinking status, obesity, cigarette consumption, and other lifestyle. Thirdly, heterogeneity is a potential problem when interpreting all the results of meta-analysis. Although we minimized the likelihood by performing a careful search for published studies, using the explicit criteria for study inclusion, the significant between-study heterogeneity still existed in most of comparison. Besides, subgroup analysis and meta-regression were also used to identify the source of heterogeneity. The presence of heterogeneity can result from differences in the age distribution, obesity status of subjects, selection of controls, dietary habits, prevalence lifestyle factors and so on. Last but not least, only published studies were included in this meta-analysis. Therefore, publication bias may have occurred, even though the use of a statistical test did not show it.

To conclude, this meta-analysis showed that the *MTNR1B* rs10830963 polymorphism was significantly associated with increased risk of T2D, particularly in the Caucasian population. In addition, our meta-analysis results suggest that *MTNR1B* rs10830963 is risk factor for the development of impaired glucose regulation. Moreover, gene–gene and gene–environment interactions should be considered in future studies.

## Supporting Information

Checklist S1 (DOC)

Figure S1 **Study selection process.**
(TIF)

Figure S2 **Result of sensitivity analyses for** ***MTNR1B*** **rs10830963.**
(TIF)

Figure S3 **Result of sensitivity analyses for** ***MTNR1B*** **rs1387153.**
(TIF)

Figure S4 **Begg's funnel plot of** ***MTNR1B*** **rs10830963 polymorphism and T2D risk.**
(TIF)

Figure S5 **Begg's funnel plot of** ***MTNR1B*** **rs1387153 polymorphism and T2D risk.**
(TIF)

Figure S6 **Test publication bias of studies of the rs10830963 polymorphism of** ***MTNR1B*** **and T2D using Egger test.**
(TIF)

Figure S7 **Test publication bias of studies of the rs1387153 polymorphism of** ***MTNR1B*** **and T2D using Egger test.**
(TIF)
