# Supplementary figures and images for: Association between the Melatonin Receptor 1B Gene Polymorphism on the Risk of Type 2 Diabetes, Impaired Glucose Regulation: A Meta-Analysis
Source: PLoS One. 2012 Nov 30;7(11):e50107. doi: 10.1371/journal.pone.0050107 (PMC3511448; doi:10.1371/journal.pone.0050107)

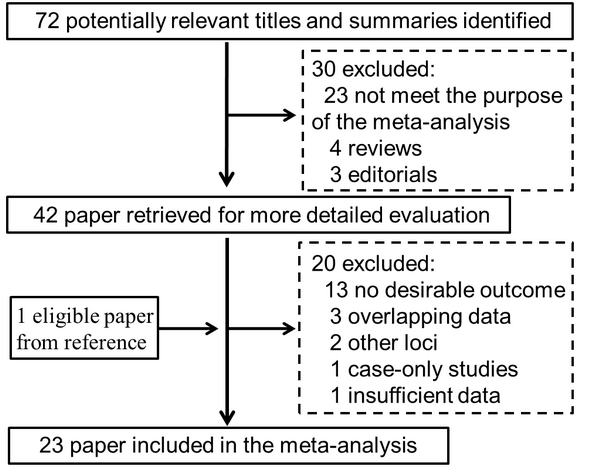

Supplement: Figure S1 — Study selection process. (TIF) [file pone.0050107.s002.tif]

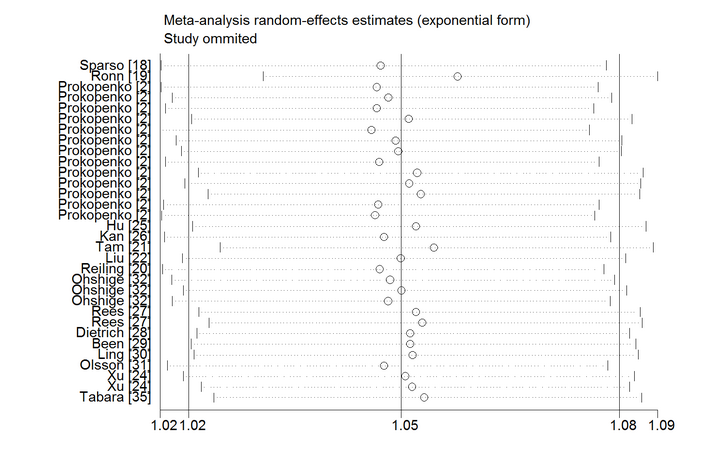

Supplement: Figure S2 — Result of sensitivity analyses for MTNR1B rs10830963. (TIF) [file pone.0050107.s003.tif]

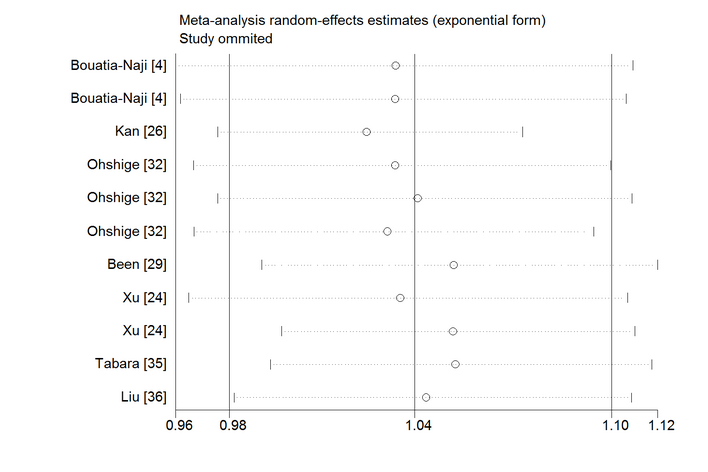

Supplement: Figure S3 — Result of sensitivity analyses for MTNR1B rs1387153. (TIF) [file pone.0050107.s004.tif]

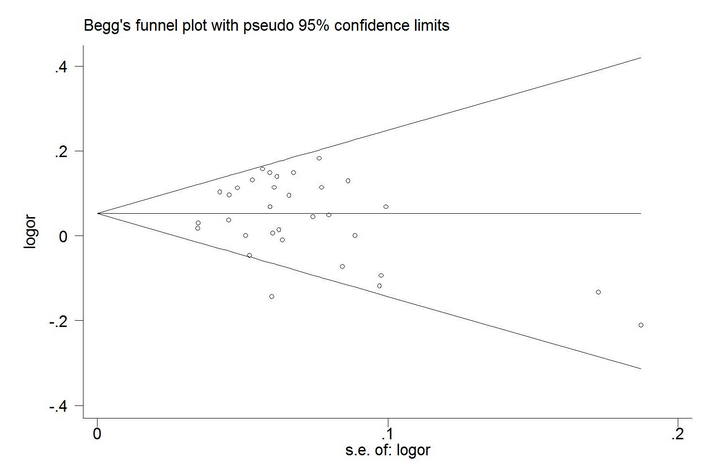

Supplement: Figure S4 — Begg's funnel plot of MTNR1B rs10830963 polymorphism and T2D risk. (TIF) [file pone.0050107.s005.tif]

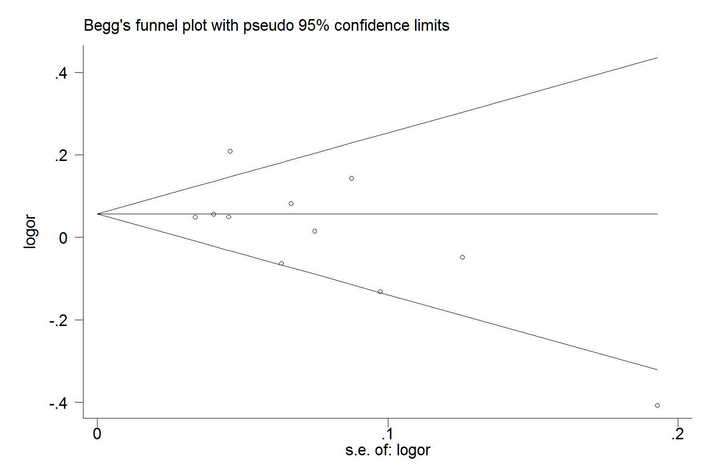

Supplement: Figure S5 — Begg's funnel plot of MTNR1B rs1387153 polymorphism and T2D risk. (TIF) [file pone.0050107.s006.tif]

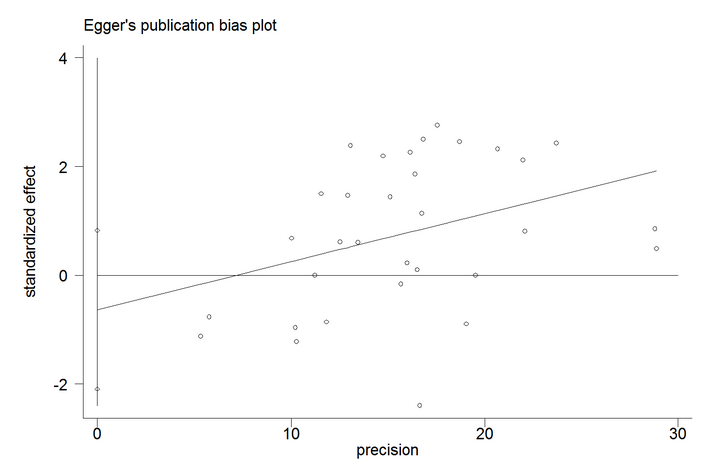

Supplement: Figure S6 — Test publication bias of studies of the rs10830963 polymorphism of MTNR1B and T2D using Egger test. (TIF) [file pone.0050107.s007.tif]

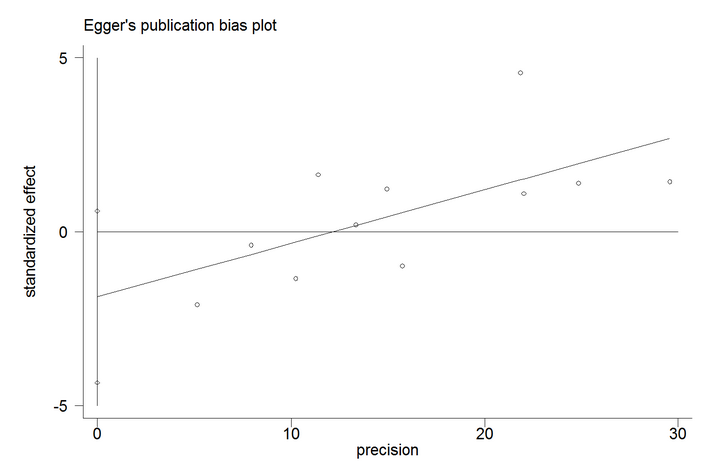

Supplement: Figure S7 — Test publication bias of studies of the rs1387153 polymorphism of MTNR1B and T2D using Egger test. (TIF) [file pone.0050107.s008.tif]
